# Milling Positive Master for Polydimethylsiloxane Microfluidic Devices: The Microfabrication and Roughness Issues

**DOI:** 10.3390/mi8100287

**Published:** 2017-09-21

**Authors:** Zhizhi Zhou, Dong Chen, Xiang Wang, Jiahuan Jiang

**Affiliations:** Key Laboratory of Biorheological Science and Technology, Ministry of Education, Bioengineering College of Chongqing University, Chongqing 400044, China; dzhizhou@cqu.edu.cn (Z.Z.); dongch028@cqu.edu.cn (D.C.); xwangchn@cqu.edu.cn (X.W.)

**Keywords:** microchannel, microfluidic, milling, acrylic, emulsions, polydimethylsiloxane (PDMS)

## Abstract

We provide a facile and low-cost method (F-L) to fabricate a two-dimensional positive master using a milling technique for polydimethylsiloxane (PDMS)-based microchannel molding. This method comprises the following steps: (1) a positive microscale master of the geometry is milled on to an acrylic block; (2) pre-cured PDMS is used to mold the microscale positive master; (3) the PDMS plate is peeled off from the master and punctured with a blunt needle; and (4) the PDMS plate is O_2_ plasma bonded to a glass slide. Using this technique, we can fabricate microchannels with very simple protocols quickly and inexpensively. This method also avoids breakage of the end mill (ϕ = 0.4 mm) of the computerized numerical control (CNC) system when fabricating the narrow channels (width < 50 µm). The prominent surface roughness of the milled bottom-layer could be overcomed by pre-cured PDMS with size trade-off in design. Finally, emulsion formation successfully demonstrates the validity of the proposed fabrication protocol. This work represents an important step toward the use of a milling technique for PDMS-based microfabrication.

## 1. Introduction

Numerous microfluidic applications [1,2] have evolved due to the introduction of soft lithography in the late 1990s, which is a more accessible microfabrication strategy [3]. In soft lithography, an elastomeric material—typically polydimethylsiloxane (PDMS)—is cast against a positive, pre-formed relief master, then the cured PDMS block is peeled off for further sealing of the microchannels and placement of the tubing, finally forming a microfluidic prototype device. This soft prototyping method is less expensive and much faster than most conventional microfabrication techniques; however, it still suffers from several drawbacks—the most important being that photolithography is used to produce the master, which typically requires an expensive clean-room facility. Many efforts have been made toward master fabrication using non-photolithography to reduce costs, increase speed, and ease processing, largely by avoiding the need for expensive facilities. One method is based on toner [4], and uses laser or inkjet printers to print micron-sized features of toner wax or ink, which can be used directly [5,6,7,8,9,10,11,12,13,14] or indirectly (or as a sacrifice template) [15,16,17,18] to form microchannel structures. Among these, the method for etching flexible copper printed circuit boards [13], “Shrinky-Dink” process [17], and ink-sacrificial template [18] have made this approach a little more flexible for changing the features of microchannels. While toner-based fabrication methods can meet a range of technical needs, there are still challenges in the area of polymers (e.g., PDMS-based microfluidic prototyping).

As an alternative method, micromilling has the potential to address some of these challenges [19]. Micromilling is a fabrication method that creates microscale features via cutting tools that remove bulk material. Modern computerized numerical control (CNC) mills are versatile and capable of fabricating devices with features just several microns in size [20]. This technology has already been utilized to make microdevices; for example, directly for microfluidic applications [21,22,23,24,25,26], and indirectly for molds used in subsequent fabrication steps such as embossing or injection molds [27,28]. More recently, Carugo et al. [29] developed a technique combining micromilling-replica molding (µMi-REM) to fabricate the negative poly (methyl methacrylate) (PMMA) master for microscale PDMS architectures. Their proposed method could allow for rapid (~100 min) production of the master layer in conventional mechanical milling workshops, but requires double molding and de-molding with an epoxy adhesive as the intermediate molding material. However, the method remains a barrier in broad applications, partly because of presumed high start-up costs, the need for large equipment and lab space, and the need for extensive technical expertise. Wu et al. [30] introduced a positive PMMA master for negative PDMS-based microarchitecture molding in a biological application. However, the size of microchannels in their work was on the scale of a millimeter. Furthermore, common milling fabrication on plastic polymers with low stiffness (e.g., PMMA) indeed introduces unavoidable surface roughness, which highlights the need for a better solution.

In this paper, we attempt to address some of the above limitations to create a facile method for producing PDMS-based microfluidic devices using a strategy similar to the above-mentioned µMi-REM [29] that combines common mechanical micromilling and replica molding [29]. Different from the work of Carugo et al. [29], in our protocol we fabricate a positive 2D micropattern directly in PMMA rather than a negative one as the master mold for subsequent molding of a PDMS replica. Then, we apply a step of partial cure of PDMS prepolymer to enhance the surface relief replica from the positive master to cured PDMS. Compared with the fabrication of patterns in negative masters via mechanical-milling, that of a positive master uses only one end mill to complete, and only a single molding step which is simpler and results in reduce time for the construction of PDMS microdevices. 

## 2. Materials and Methods

### 2.1. Materials

Polydimethylsiloxane (Sygard 184 silicone Elastomer Kit), Span 80, and silicone oil with a viscosity of 50 mPa s were purchased from Dow Corning Corporation (Midland, MI, USA). Common PMMA sheets or acrylic plates with thickness 4 mm were purchased from Chengdu Guangxinhe Trade Company (Chengdu, China); PTFE (polytetrafluoroethylene) tubes of inner diameters 0.35 mm were from Woer Heat-Shrinkable Material Co., Ltd. (Shenzhen, China).

### 2.2. Protocol of the Fabrication of PDMS Microchannel Device through Milling Positive Master and Molding

**Figure 1:**
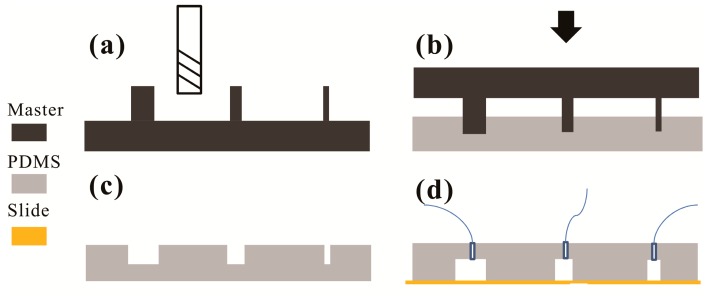
Schematic illustration of polydimethylsiloxane (PDMS)-based microchannel fabrication based on replica positive master created using a milling technique. (**a**) The positive master is mechanically milled in an acrylic block; (**b**) The positive micropatterns are inserted into pre-cured PDMS agent; (**c**) The solidified PDMS block is peeled off the master; (**d**) The PDMS block is bonded onto a glass plate by O_2_ plasma treatment.

The protocol of microchannel fabrication using positive master molding is as follows (Figure 1):

(i) The geometrical pattern of the microchannels is designed using computer-aided design software such as Autodesk (AutoCAD 2017, Autodesk, Inc., San Rafael, CA, USA).
(ii) The convex of the pattern is milled on a PMMA sheet using a CNC mini-engraving machine (custom-made based on Tonsen CNC (Dongda Electrical Control Company, Jiangyin, China) and see also below) with an end-mill (ϕ = 0.4 mm, double-edged, tungsten steel) for the positive master (Figure 1a), which is to be used in the subsequent steps.
(iii) The block of the above positive master is cleaned with mild detergents, and washed in pure water for 15 min with ultrasonic, and then flushed dry.
(iv) Liquid PDMS mixture (prepolymer: curing agent = 10:1 *w*/*w*) is poured into a dish to the predefined depth, then degassed for ~10 min, and pre-cured for 30 min at 55 °C in an oven. Then, on the top surface of the pre-cured PDMS the cleaned positive master block is covered with the convex pattern top-down, and the whole dish is placed in the oven for curing again 90 min at 55 °C (Figure 1b).
(v) After curing, the block of the positive master is removed, leaving the solidified PDMS block with the negative pattern (Figure 1c). This PDMS block is then bonded onto a glass slide via O_2_ plasma treatment (O_2_ pressure 0.1 MPa, power 25 W, processing time 10–20 s, Diener Prep2, Diener electronic GmbH, Ebhausen, Germany) (Figure 1d).

## 3. Results and Discussions

### 3.1. 2D Positive Master Fabrication

**Figure 2:**
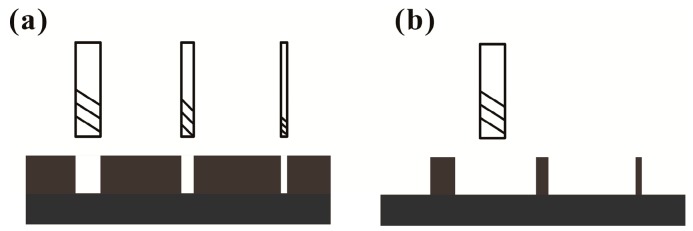
Advantage of positive master. (**a**) Different width of negative master milled by variable dimension of end mill; (**b**) Different width of positive master milled by only one dimension of end mill.

In this work, a protocol for facile and low-cost (F-L) fabrication of a PDMS microchannel device was developed. This protocol has two critical points: one is the use of a low-cost CNC milling machine to prepare the two-dimensional (2D) positive master in a PMMA block for subsequently establishing a PDMS microfluidic device; the other is using only a single molding step in the pattern transfer. The benefits of using a positive master in the making of a microfluidic device could have several points. It could obviously reduce the molding steps from double to single, as the positive master could directly transfer its convex surface into the real channel after molding, while the negative cannot [29]. It could produce fine channel-structure in the milling process. Specifically, the milling is intrinsically a subtractive process; this characteristic feature could make the width of a convex surface (acting as the channel master) much more fine when given milling from its two opposite sides. Moreover, compared with most negative master-based techniques (Figure 2a), it could use only one end-mill to fabricate channels with different width (Figure 2b); nevertheless, the geometric size of the cross-junction between two channels may be limited.

In order to make the protocol low-cost and more accessible, we seek to employ a CNC machine with a low price. In this work, the CNC mini-engraving machine was custom-made based on Tonsen CNC. The main specifications of the CNC mini-engraving machine were supplied in Table 1, according to the manufacturer. When equipped with a common small crane-mill, this mini-engraving machining met our fabrication needs. 

However, the main concern is the issue of surface roughness when using a low-end milling machine in the fabrication of microdevices. Indeed, roughness will unavoidably occur in the milling process, even in machines with higher precision. Fortunately, the adoption of a positive master for the template for molding microchannels reduces such difficulty, as the convex surface of the positive master is the natural smooth surface of the PMMA block, after molding the thus-obtained bottom of channel is certain to be equally smooth. So, we next focus on how to reduce the roughness on the bottom-walls of the molded channels.

After testing the fabrication potential of the channel’s minimum width when using the positive strategy, we observed the results when adopting several diameters of end mill. Table 2 is a data comparison between our work and references. We found that we could employ a large end mill (ϕ = 0.4 mm) to fabricate our microchannels with width as small as 50 µm. Table 3 lists several main features as a result of adopting the two respective types of master (i.e., negative and positive) in the microchannel molding. Compared to negative master-based microchannel fabrication, our F-L method shows several good features from observing their flexibility, convenience, and stabilization. In addition, the time spent in our positive-based protocol is about 3 h—a great reduction when compared with that of Ref. [29] (Table 4).

### 3.2. Measurement of Microchannels

**Figure 3:**
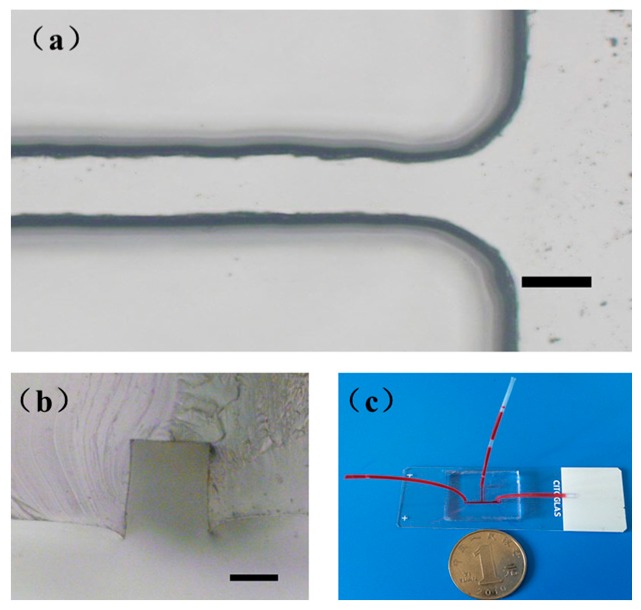
(**a**) Representative microscopic images of microchannels fabricated using a milling technique, captured with microscopy (IX71, Olympus Optical Co., Tokyo, Japan); (**b**) A cross-sectional view of microchannel depicted in (**a**); Scale bars indicate 50 μm in both (**a**,**b**). (**c**) A photograph of the prototype microchip with red ink in the negative microchannels bonded to a glass slide.

In general, PDMS microchannel devices fabricated based on negative master require double casting and de-molding [27,29,32]. As mentioned above, in this study, a PDMS replica with negative microchannels could only be realized in a single molding step from a positive master (Figure 1 and Figure 3). Figure 3a shows the representative microscopic image of PDMS microchannels at one part of the microdevices created using this positive master mold. Figure 3b shows the microscopic image of the cross-section of the PDMS microchannels. Figure 3c shows the picture of the microdevice after bonding. The sizes of microchannels in microscopy (IX71, Olympus Optical Co., Tokyo, Japan) images of channels were measured using ImageJ software (NIH, Rockville, MD, USA).

In order to generate a good profile of the microchannels, the depth of the milled positive microstructure (depth ≥ 200 µm) should be larger than the molding channel (depth ≤ 100 µm); this could ensure that the partial curing agent contacted the milled roughness surface; meanwhile, the thickness of the PDMS layer should also be larger than the channels. Therefore, we need to form a PDMS layer which is about 4 mm thick in the subsequent steps. Table 5 shows the standard deviation of the width and depth in this protocol. A slight reduction in the average aspect ratio (width/depth) of channels was shown in the PDMS bonding step (i.e., 1.94–1.58). Due to the addition of the pre-curing process step before PDMS molding in the protocol, side-crawling was induced by capillary action, and there was a slight roll-up around the side of the microchannels (i.e., 8.5 ± 3.0 µm). However, this drawback is not an obstacle in the production of microdevices using this method.

### 3.3. Atomic Force Microscopy (AFM) and Profilometer Images of Surface Roughness Analysis

The surface quality of milled blocks could be fine-tuned by several factors in the CNC milling system, including machine precision, spindle speed, feed rate, and depth of cut [19,33]. However, the surface polishing process for the milled acrylic block is still a challenging problem. Some researchers have used a chloroform solvent vapor polishing technique for PMMA and cyclic olefin copolymer [34]; however, this method needs additional cost for surface modification.

**Figure 4:**
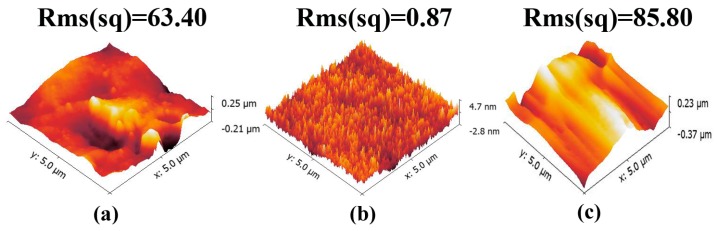
(**a**) Atomic force microscopy (AFM) images on the surface of the milled layer onto PDMS-molding; (**b**) AFM images on the surface of the pre-cured PDMS; (**c**) AFM images on the surface of the side wall.

**Figure 5:**
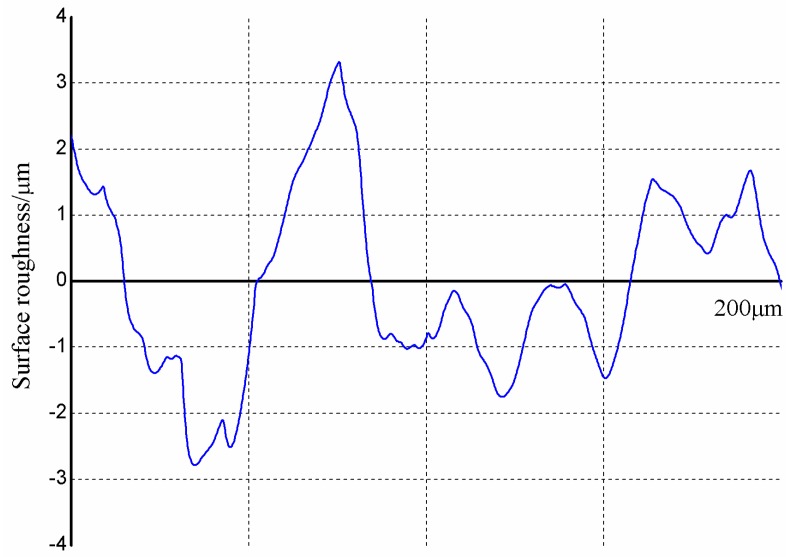
Surface roughness of the side wall in milled PDMS microchannels.

To overcome these drawbacks, we controlled the crosslinking state of PDMS by adjusting the time and temperature during the pre-curing process to prevent the capillary crawling of liquid PDMS, and then inserted the master microstructures into the pre-curing PDMS to avoid the contact between the cured PDMS top surface and the bottom surface of the acrylic block milled by the end mill (Figure 1b). Figure 4 shows the AFM analysis results of the milled layer onto PDMS molding (Figure 4a) and the pre-cured PDMS layer (Figure 4b) in the square of 5 µm × 5 µm. It could be observed that the milled block with an average surface roughness value (*R*_a_) of 6.34 × 10^2^ µm could be transferred to the PDMS layer with 0.87 × 10^3^ µm of average surface roughness value (*R*_a_). The pre-cured PDMS layer provides a better surface for PDMS bonding to glass slide. However, in our proposed method, there are also some limitations which are similar to other techniques. For example, we are not able to make those channels closer together than the diameter of the bit. Surface roughness of the side wall (Figure 4c) seems to be high in comparison with the information supplied in Figure 4a,b. Moreover, Figure 5 shows the roughness (*R*_a_ = 2.5 µm) profile of the side wall measured with the profilometer (Dektak 150, Veeco instruments Inc., Town of Oyster Bay, New York, NY, USA), wherein the curves fluctuation and the parameters (cutoff of 200 µm and six samples) can be seen. The need to achieve accurate surfaces for fluid transportation is challenging, but it is not a barrier to use in the formation of emulsions.

To demonstrate the utility of our PDMS devices using this technique, we used the devices to produce emulsions in different flow patterns in the T-shape microstructure. Three PTFE tubes were inserted into two inlets and one outlet of the chip. Two syringe pumps (LSP10-1B, Baoding Longer Precision Pump Co., Ltd., Baoding, China) were used to inject water (dispersed phase) and oil (continuous phase) into the two inlets, respectively. The inner dispersed phase was deionized water. Silicone oil was used as the continuous phase with 10 wt % Span 80.

**Figure 6:**
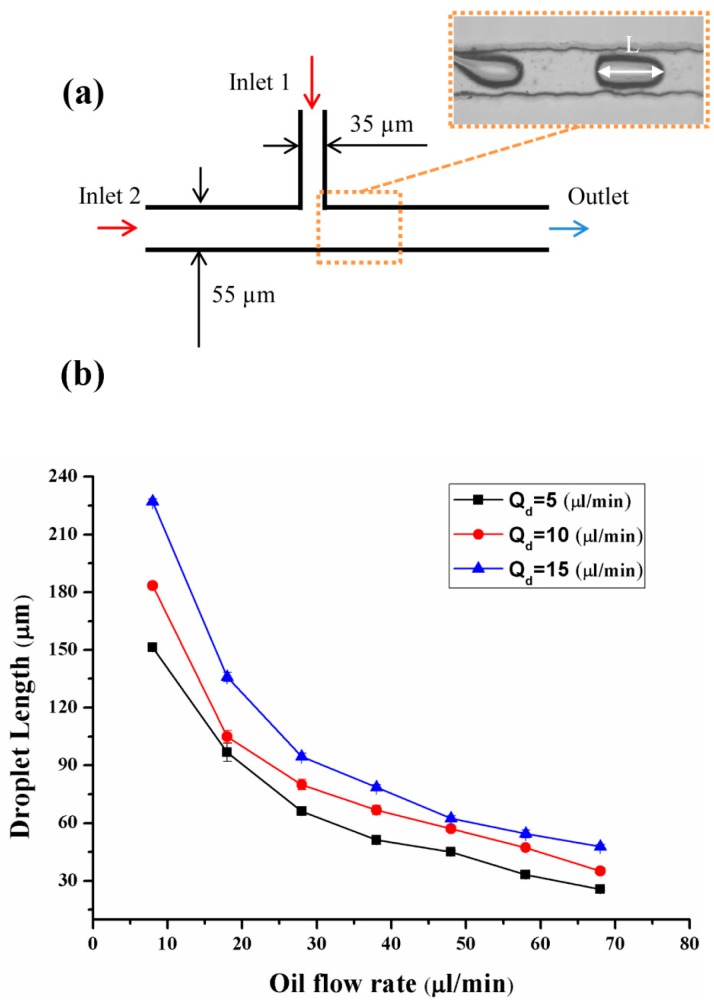
(**a**) The design of the “T-junction” microdevice used for generating emulsions. All channels have a depth of 50 µm. The inset illustrates the generation of the water-in-oil emulsions in the device. The parameter “L” represents the length of a droplet; (**b**) Relationship between the droplet length and flow rate of oil and water in the two-dimensional “T-shape” junction microchannel. Q_d_ is a parameter of the dispersed phase (deionized water) flow rates. The curve fits were obtained using experimental data.

Representative microscopic images of the droplets obtained are shown in the inset of Figure 6a. We studied the droplets formed in the “T-shape” microchannels by varying the flow rate of the dispersed phase and continuous phase. For each condition, the length of 50 droplets was measured, and the coefficient of variation was 4% or lower. Figure 6b shows the correlation between the length of droplets and flow rate in each condition. 

## 4. Conclusions

We present a more facile and low-cost method for the construction of PDMS microfluidic devices through milling a positive master and a minimum casting step. Here, the thus-fabricated acrylic positive pattern could be a good template to produce PDMS microchannel devices. We demonstrated the ability of the thus-prepared microchannel device in emulsion generation. Though the surface roughness of the side walls seems to require further improvements, the method demonstrated here will nevertheless provide a platform on which researchers could quickly develop prototypes of microfluidic devices for droplet formation and other applications.

## Figures and Tables

**Table 1:** Computerized numerical control (CNC) mini-engraving machine specifications.

Axis Travel	Parameter
*X*-, *Y*-, and *Z*-axes travel (mm)	180 × 220 × 30
Table working area (mm)	270 × 350
Table weight (kg)	17
Feed per tooth (fz)	0.04
Spindle motor (kw)	0.2
Spindle speed (rpm)	0–2000
Cutting feed rate (mm/min)	0–3000
Position precision (mm)	0.05
Position Repeatability (mm)	0.02
Control	TS-2518C
Lubrication	No use

**Table 2:** Minimum width of positive microchannel milled by different diameter mills, and comparison with minimum width of negative microchannel by micromilling.

Diameter of Mills (in mm)	Minimum Width (in mm)	Reference
~1	~1	[27]
0.1	~0.1	[29]
0.03	~0.03	[31]
0.4	~0.03	This work

**Table 3:** Comparison of the features between positive master and negative master for PDMS molding.

Types of Master	End Mill	Replica Time	Roughness Surface	Microstructure	Ref.
Negative	Several	Twice	Side and bottom	Dependent on mill size	[29]
Positive	Only one	Once	Side	Independent on mill size (except cross-junction)	This work

**Table 4:** Comparison of the time consumed between positive master and negative master in every main step.

Types of Master	Master Fabrication	PDMS Moulding	Ref.
Negative	~100 min	Over night	[29]
Positive	~30 min	120 min	This work

**Table 5:** The average and standard deviation (*n* = 3) of microchannel width and depth measured after every step.

Positive Master	PDMS	PDMS after Bonding
**Width (µm)**		
105.6 ± 5.2	107.3 ± 7.6	95.5 ± 8.5
**Depth (µm)**		
-	55.2 ± 3.8	60.3 ± 5.8

## References

[1] Whitesides G.M. (2006). The origins and the future of microfluidics. Nature.

[2] Sackmann E.K., Fulton A.L., Beebe D.J. (2014). The present and future role of microfluidics in biomedical research. Nature.

[3] Xia Y.N., Whitesides G.M. (1998). Soft lithography. Angew. Chem. Int. Ed..

[4] Tomazelli Coltro W.K., de Jesus D.P., Fracassi da Silva J.A., do Lago C.L., Carrilho E. (2010). Toner and paper-based fabrication techniques for microfluidic applications. Electrophoresis.

[5] Tan A.M., Rodgers K., Murrihy J.P., O’Mathuna C., Glennon J.D. (2001). Rapid fabrication of microfluidic devices in poly(dimethylsiloxane) by photocopying. Lab Chip.

[6] Branham M.L., Tran-Son-Tay R., Schoonover C., Davis P.S., Allen S.D., Shyy W. (2002). Rapid prototyping of micropatterned substrates using conventional laser printers. J. Mater. Res..

[7] Do Lago C.L., da Silva H.D.T., Neves C.A., Brito-Neto J.G.A., da Silva J.A.F. (2003). A dry process for production of microfluidic devices based on the lamination of laser-printed polyester films. Anal. Chem..

[8] Coltro W.K.T., da Silva J.A.F., da Silva H.D.T., Richter E.M., Furlan R., Angnes L., do Lago C.L., Mazo L.H., Carrilho E. (2004). Electrophoresis microchip fabricated by a direct-printing process with end-channel amperometric detection. Electrophoresis.

[9] Bao N., Zhang Q., Xu J.J., Chen H.Y. (2005). Fabrication of poly(dimethylsiloxane) microfluidic system based on masters directly printed with an office laser printer. J. Chromatogr. A.

[10] Liu A.L., He F.Y., Wang K., Zhou T., Lu Y., Xia X.H. (2005). Rapid method for design and fabrication of passive micromixers in microfluidic devices using a direct-printing process. Lab Chip.

[11] Vullev V.I., Wan J.D., Heinrich V., Landsman P., Bower P.E., Xia B., Millare B., Jones G. (2006). Nonlithographic fabrication of microfluidic devices. J. Am. Chem. Soc..

[12] Kaigala G.V., Ho S., Penterman R., Backhouse C.J. (2007). Rapid prototyping of microfluidic devices with a wax printer. Lab Chip.

[13] Abdelgawad M., Watson M.W.L., Young E.W.K., Mudrik J.M., Ungrin M.D., Wheeler A.R. (2008). Soft lithography: Masters on demand. Lab Chip.

[14] Lobo Júnior E.O., Duarte L.C., Braga L.E.P., Gobbi A.L., de Jesus D.P., Coltro W.K.T. (2015). High fidelity prototyping of PDMS electrophoresis microchips using laser-printed masters. Microsyst. Technol..

[15] Do Lago C.L., Neves C.A., de Jesus D.P., da Silva H.D.T., Brito-Neto J.G.A., da Silva J.A.F. (2004). Microfluidic devices obtained by thermal toner transferring on glass substrate. Electrophoresis.

[16] Coltro W.K.T., Piccin E., da Silva J.A.F., do Lago C.L., Carrilho E. (2007). A toner-mediated lithographic technology for rapid prototyping of glass microchannels. Lab Chip.

[17] Grimes A., Breslauer D.N., Long M., Pegan J., Lee L.P., Khine M. (2008). Shrinky-Dink microfluidics: Rapid generation of deep and rounded patterns. Lab Chip.

[18] Guo Y.Z., Li L.H., Li F.Y., Zhou H.H., Song Y.L. (2015). Inkjet print microchannels based on a liquid template. Lab Chip.

[19] Guckenberger D.J., de Groot T.E., Wan A.M.D., Beebe D.J., Young E.W.K. (2015). Micromilling: A method for ultra-rapid prototyping of plastic microfluidic devices. Lab Chip.

[20] Aurich J.C., Reichenbach I.G., Schuler G.M. (2012). Manufacture and application of ultra-small micro end mills. Cirp Ann. Manuf. Technol..

[21] Berthier E., Guckenberger D.J., Cavnar P., Huttenlocher A., Keller N.P., Beebe D.J. (2013). Kit-On-A-Lid-Assays for accessible self-contained cell assays. Lab Chip.

[22] Guckenberger D.J., Berthier E., Beebe D.J. (2015). High-Density Self-Contained Microfluidic KOALA Kits for Use by Everyone. J. Lab. Autom..

[23] Casavant B.P., Guckenberger D.J., Berry S.M., Tokar J.T., Lang J.M., Beebe D.J. (2013). The VerIFAST: An integrated method for cell isolation and extracellular/intracellular staining. Lab Chip.

[24] Strotman L., O’Connell R., Casavant B.P., Berry S.M., Sperger J.M., Lang J.M., Beebe D.J. (2013). Selective Nucleic Acid Removal via Exclusion (SNARE): Capturing mRNA and DNA from a Single Sample. Anal. Chem..

[25] Bischel L.L., Mader B.R., Green J.M., Huttenlocher A., Beebe D.J. (2013). Zebrafish Entrapment by Restriction Array (ZEBRA) device: A low-cost, agarose-free zebrafish mounting technique for automated imaging. Lab Chip.

[26] Carney C.M., Muszynski J.L., Strotman L.N., Lewis S.R., O’Connell R.L., Beebe D.J., Theberge A.B., Jorgensen J.S. (2014). Cellular Microenvironment Dictates Androgen Production by Murine Fetal Leydig Cells in Primary Culture. Biol. Reprod..

[27] Wilson M.E., Kota N., Kim Y., Wang Y.D., Stolz D.B., LeDuc P.R., Ozdoganlar O.B. (2011). Fabrication of circular microfluidic channels by combining mechanical micromilling and soft lithography. Lab Chip.

[28] Okagbare P.I., Emory J.M., Datta P., Goettert J., Soper S.A. (2010). Fabrication of a cyclic olefin copolymer planar waveguide embedded in a multi-channel poly(methyl methacrylate) fluidic chip for evanescence excitation. Lab Chip.

[29] Carugo D., Lee J.Y., Pora A., Browning R.J., Capretto L., Nastruzzi C., Stride E. (2016). Facile and cost-effective production of microscale PDMS architectures using a combined micromilling-replica moulding (μMi-REM) technique. Biomed. Microdevices.

[30] Wu H.W., Lin C.C., Hwang S.M., Chang Y.J., Lee G.B. (2011). A microfluidic device for chemical and mechanical stimulation of mesenchymal stem cells. Microfluid. Nanofluid..

[31] Singhal J., Pinho D., Lopes R., Sousa P.C., Garcia V., Schütte H., Lima R., Gassmann S. (2015). Blood Flow Visualization and Measurements in Microfluidic Devices Fabricated by a Micromilling Technique. Micro Nanosyst..

[32] Gitlin L., Schulze P., Belder D. (2009). Rapid replication of master structures by double casting with PDMS. Lab Chip.

[33] Chen P.C., Pan C.W., Lee W.C., Li K.M. (2014). An experimental study of micromilling parameters to manufacture microchannels on a PMMA substrate. Int. J. Adv. Manuf. Technol..

[34] Ogilvie I.R.G., Sieben V.J., Floquet C.F.A., Zmijan R., Mowlem M.C., Morgan H. (2010). Reduction of surface roughness for optical quality microfluidic devices in PMMA and COC. J. Micromech. Microeng..

